# “Black box warning” rash with entecavir - case report

**DOI:** 10.1186/s12876-020-01452-3

**Published:** 2020-09-18

**Authors:** Xiong Khee Cheong, Zhiqin Wong, Norazirah Md Nor, Bang Rom Lee

**Affiliations:** 1 Department of Medicine, Faculty of Medicine, Universiti Kebangsaan Malaysia, Jalan Yaacob Latif, Bandar Tun Razak, 56000 Kuala Lumpur, Malaysia; 2 Gastroenterology and Hepatology Unit, Department of Medicine, Faculty of Medicine, Universiti Kebangsaan Malaysia, Jalan Yaacob Latif, Bandar Tun Razak, 56000 Kuala Lumpur, Malaysia; 3 Dermatology Unit, Department of Medicine, Faculty of Medicine, Universiti Kebangsaan Malaysia, Jalan Yaacob Latif, Bandar Tun Razak, 56000 Kuala Lumpur, Malaysia; 4 Department of Pathology, Hospital Pantai Kuala Lumpur, Jalan Bukit Pantai, Bangsar, 59100 Kuala Lumpur, Malaysia

**Keywords:** Drug eruption, Entecavir, Hepatitis B

## Abstract

**Background:**

Hepatitis B infection is a significant worldwide health issue, predispose to the development of liver cirrhosis and hepatocellular carcinoma. Entecavir is a potent oral antiviral agent of high genetic barrier for the treatment of chronic hepatitis B infection. Cutaneous adverse reaction associated with entecavir has rarely been reported in literature. As our knowledge, this case was the first case reported on entecavir induced lichenoid drug eruption.

**Case presentation:**

55 year old gentlemen presented with generalised pruritic erythematous rash on trunk and extremities. Six weeks prior to his consultation, antiviral agent entecavir was commenced for his chronic hepatitis B infection. Skin biopsy revealed acanthosis and focal lymphocytes with moderate perivascular lymphocyte infiltration. Skin condition recovered completely after caesation of offending drug and short course of oral corticosteroids.

**Conclusion:**

This case highlight the awareness of clinicians on the spectrum of cutaneous drug reaction related to entecavir therapy.

## Background

Hepatitis B infection is a global health issue, contributing to approximately 887,000 deaths in 2015 due to hepatocellular carcinoma and liver cirrhosis [1, 2]. Entecavir is a nucleoside analogue reverse transcriptase inhibitor that is widely used in the treatment of chronic hepatitis B (HBV) infection. Adverse events associated with entecavir that commonly reported were headache, fatigue, myalgia, dizziness, nausea, raise alanine transaminase (ALT) and lactic acidosis. Cutaneous adverse reactions is a rare complication, only reported in few case reports. A case of lichenoid drug eruption associated with entecavir therapy has been described here for its rarity and unusual adverse effect.

## Case presentation

**Fig. 1:**
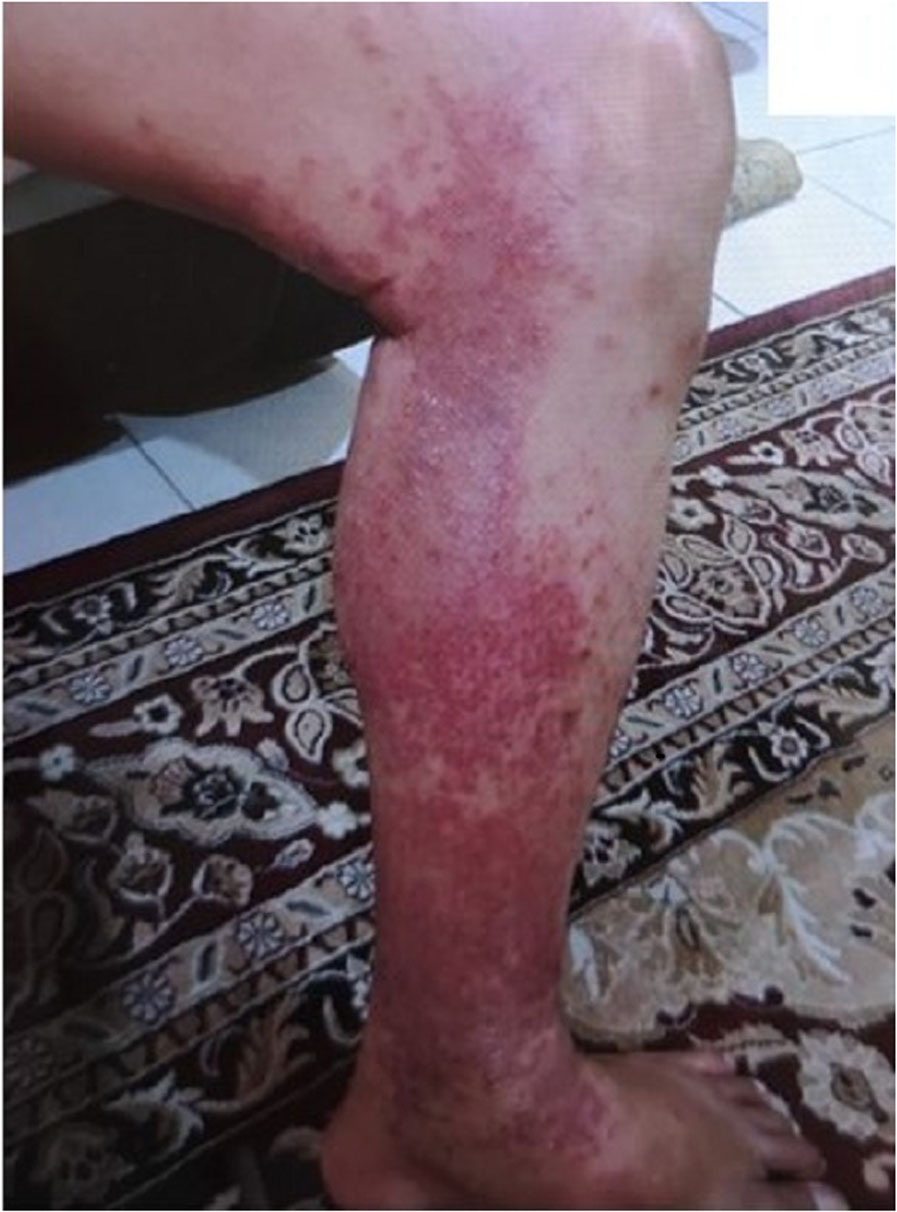
Pruritic erythematous patches with scaly on right lower limb

A 55 years old gentlemen, background of chronic hepatitis B (treatment naïve), was referred for 2 weeks of generalised itchy erythematous rash. His other medical illnesses were ischemic heart disease, chronic kidney disease stage 3, diabetes mellitus and hypertension of which he has been on a stable medication for one year with no recent alteration of medications except for initiation of treatment for hepatitis B. Entecavir 0.5 mg once in 48 h was initiated 2 months ago in view of fibroscan revealed significant liver fibrosis with eGFR ranging between 30 and 49 mL/min/1.73m^2^. Four weeks after initiation of entecavir therapy, he developed multiple pruritic erythematous patches started on bilateral legs and gradually spread to lower thighs and trunk. The patient did not show other systemic symptoms. On examination, there was extensive erythematous patches with scaly edges involving the lower limbs and trunk. The face, palms and soles and the oral mucosa were spared (Fig. 1). There was no associated lymphadenopathy and other systemic examinations were normal. Laboratory investigations revealed normal leucocyte count (8.5 × 10^9^/L) with elevated absolute eosinophils count of 1.0 × 10^9^/L and raised C-reactive protein of 8.29 mg/dL (normal range ≤ 0.5). Liver function test was normal and renal profile similar with baseline, creatinine of 158 mmol/L.

**Fig. 2:**
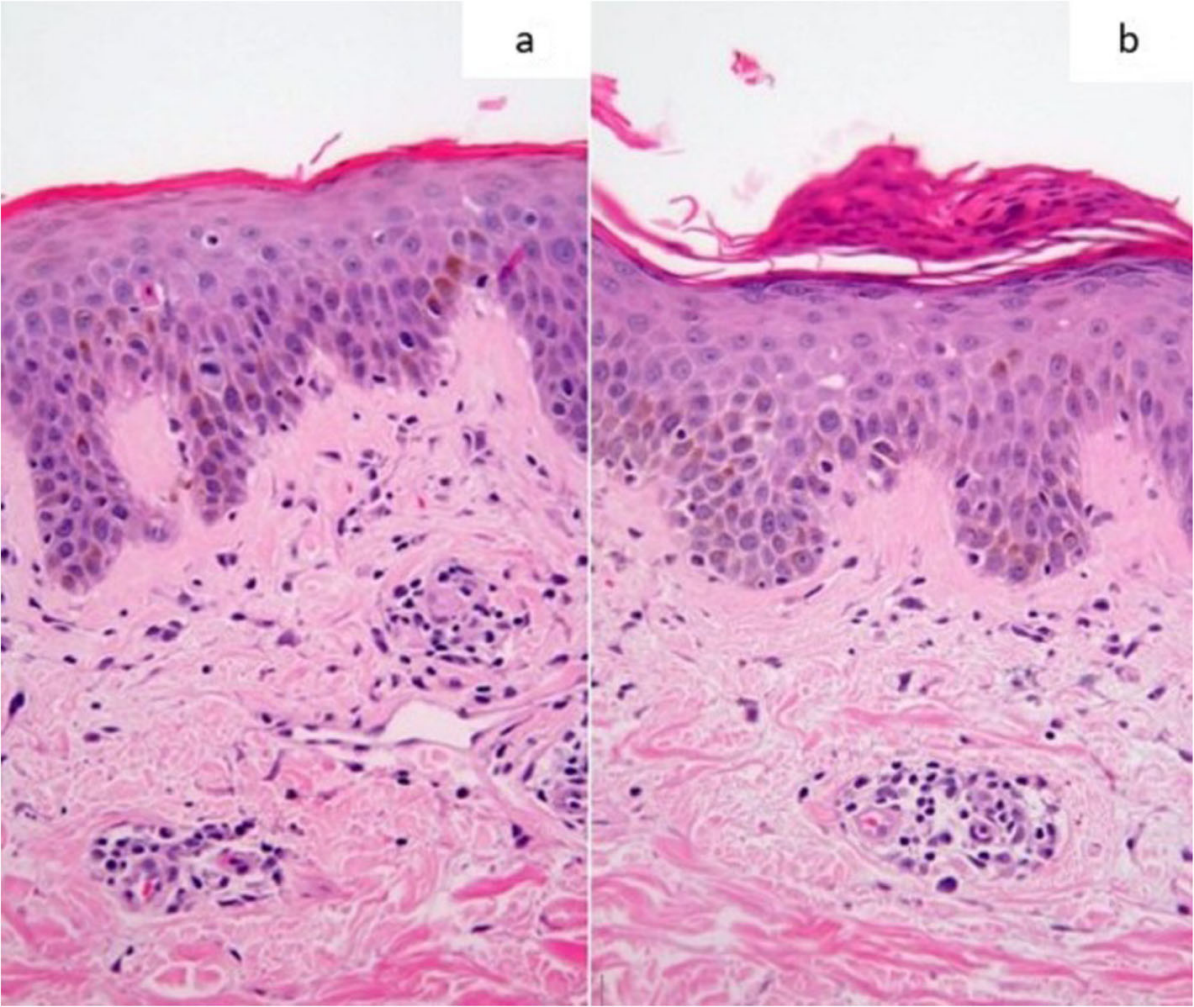
**a** & **b**: Mild acanthosis and focal parakeratosis in the epidermis. Lymphocytic inflammatory infiltrate at dermal interface and perivascular lymphocytic infiltration with occasional eosinophils at the dermis (Hematoxylin and eosin stain × 200 magnification)

Skin biopsy showed acanthosis and focal lymphocytes with dyskeratotic cells in the epidermis. There were moderate perivascular lymphocyte infiltration and occasional eosinophils at the dermis (Fig. 2a and b); no extravasation of red blood cell or vasculitic features noted. Skin patch or prick test was not performed for this patient. The patient was diagnosed with lichenoid drug eruption secondary to entecavir based on the clinical and histopathological findings.

Entecavir was discontinued immediately. The patient was started on oral corticosteroids at dose of 30 mg/day oral prednisolone and topical emollients. The lesions improved remarkably after two weeks of caseation of entecavir and initiation of oral corticosteroid. Oral prednisolone was gradually tapering off the period of over two weeks. Two months later, he was started on Tenofovir Alanfenamide 25 mg once daily for his hepatitis B treatment, he tolerated well and no further adverse events reported.

## Discussion and conclusions

Entecavir is a potent oral antiviral agent that effectively controlled chronic hepatitis B infection in adult and children. It is a nucleoside analogue that highly selective inhibitor of DNA polymerase, with a high genetic barrier towards hepatitis B virus (HBV). Clinical trials have proven that entecavir is superior to lamivudine on the treatment of drug naïve chronic hepatitis B infection by histological improvement and reduction of serum HBV DNA level [3, 4]. The commonest adverse events reported were increased ALT, lactic acidosis, headache, fatigue, dizziness, nausea and peripheral neuropathy [4].

There were limited cases published on cutaneous adverse reactions caused by entecavir (Table 1). Skin manifestation were presented in various types; from immediate allergy skin reaction [5], maculopapular rash [6, 7], granulomatous [8], bullous type [9] to erythematous patches [10]. The time interval between exposure of entecavir and the onset of symptoms varies from 2 days to 6 months.

**Table 1:** Summary on cases of drug eruption secondary to entecavir

Author/Year	Age	Sex	Clinical manifestations	Sites of lesions	Time interval^H^	DLST (drug lymphocyte stimulation test)/skin patch/scratch test	Confirmed by Histopathology
Sugiura K et al. 2009 [5]	30	Male	Anaphylaxis	Buttock	2 days	Scratch test positive	Not done
Yamada S et al. 2011 [6]	62	Male	Maculopapular rash	Trunk & extremities	7 days	DLST positive	Not done
Jimi Yoon et al. 2013 [8]	65	Female	Granulomatous	Forehead & face	2 months	Patch test negative	Yes
Maiko Taura et al. 2014 [10]	65	Male	Erythematous plaque	Upper limbs	6 months	DLST positive	Yes
Jeong Tae Kim et al. 2014 [7]	45	Male	Maculopapular rash	Back & extremities	1 months	Not done	Yes
Temiz SA et al. 2018 [9]	50	Female	Bullous eruption	Lower limb	7 days	Not done	Yes
Our case 2020	55	Male	Lichenoid erythematous patch	Trunk & extremities	6 weeks	Not done	Yes

^H^ time interval between drug exposure and onset of symptom

The recognition of the causative agent could be problematic if patient is taking more than one medication simultaneously. Skin biopsy is a useful diagnostic tool to rule out other differential diagnosis that mimic drug eruption.

To our knowledge, there were total of six cases that had been reported so far; this case is the seventh (Table 1). Our patient presented as generalised lichenoid erythematous patches, which was different from other reported cases. The diagnosis of entecavir associated drug eruption in this case was made based on the history of no recent exposure to other drugs and supported by the histopathology findings. Resolution of skin lesions upon discontinuation of the offending drug also favours the diagnosis.

Although the mechanism of action of entecavir still remains unclear, it is thought that the reduction in regulatory or helper T cells during entecavir treatment, might play a role on the development of drug eruption [11]. Besides that, the chemical structure of entecavir is similar to other nucleoside analogue antiviral agents such as lobucavir, acyclovir and ganciclovir; possibly inducing the same pattern of immunologic responses that triggered hypersensitivity skin reaction [5].

It is interesting to note that all seven cases (including our case) arise from the Asian population. The association of HLA allele and occurrence of drug eruption due to entecavir is yet to be determined. We postulate that genetic predisposition could have contribute to the hypersensitivity reaction towards entecavir.

The mainstay of treatment of drug eruption is discontinuation of the causative agent. Topical corticosteroids ointment and emollients can be use as adjunctive therapy. Systemic corticosteroids should be considered in severe or extensive cutaneous eruptions.

This case highlighted the possibility and the spectrum of cutaneous drug reaction related to entecavir. Awareness on the spectrum of cutaneous reaction and its duration of onset are important among clinician prescribing etecavir.

## Data Availability

The authors declare that all data concerning this case report are provided within the manuscript.

## Abbreviations

ALT: Alanine transaminase
HBV: Hepatitis B virus
